# Restoring the integrative value to the notion of executive function. Commentary on: “Advancing understanding of executive function impairments and psychopathology: bridging the gap between clinical and cognitive approaches”

**DOI:** 10.3389/fpsyg.2015.02040

**Published:** 2016-01-11

**Authors:** Fabián Labra-Spröhnle

**Affiliations:** Clinical Research Programme, Biology Department, Victoria University of WellingtonWellington, New Zealand

**Keywords:** executive function, functional systems, inferences, cognition, psychopathology

My comments on Snyder et al. (2015) are not targeting any particular statement contained in their review; instead they aim to highlight some overarching epistemological issues that are tacitly contained in their work and suggest an alternative standpoint.

Although a widespread mantra uttered by the majority of executive function (EF) scholars since Teuber (1972), claim the unity and diversity of the concept of EF, the search for an integrated account of the nature of EF has been as elusive as the search for its definition (Goldstein and Naglieri, 2013; Wasserman and Wasserman, 2013). These endeavors are intimately connected. A “conceptual analysis” (Deitz and Arrington, 1984; Ryle, 2009) should suffice to acknowledge the interdependence of both pursuits. Nevertheless only a few scholars have undertaken the task of assessing the epistemological grounds of EF research (Dick and Overton, 2009; Martin and Failows, 2009; Armengol de la Miyar and Moes, 2014).

To begin with, the history of the concept of EF can be seen as an instructive example of the paradoxical effects prompted by the “incommensurability” of scientific “paradigms” when looking at the same subject matter (Kuhn, 2012). In particular, the translation of theoretical notions from one paradigm to another, without assimilating the “thought style” that gave origin to them could lead to severe conceptual entanglements and disorientation (Mößner, 2011; Fleck, 2012).

The debut of a mature concept of EF was staged in Soviet dialectical materialism, migrating from there, but ill-defined, to the Anglo-Saxon cognitivist paradigm. Under this paradigm, the notion of EF was detached from its original sources i.e., (Anokhin and Bernstein's functional system theory, Vygotsky and Luria's cultural-historical approach and Filimonov's principle of “graded and pluripotential localization of functions”) (Luria, 2012), losing its integrative character and transplanted to a modular and computational view of cognition (Fodor, 1983; Newell, 1994). In this framework, cognitive functions appear like faculties, underpinned by a modular neural substrate in which the postulated EF come into sight in a more or less stable correspondence with distinct brain networks in which one is the seat of an homuncular central control (Uttal, 2001). This conceptual shift has favored multiple, *ad-hoc*, arbitrary extension to the concept of EF, with poor or null operational character without reaching consensus (Barkley, 2012). As a consequence the methodological aspect and the experimental expression of EF models are downgraded in their validity and utility.

My suggestion, to restore the integrative nature of EF, is to re-connect the concept with its overlooked origins and complement them with like theories, for example: Piaget's Constructivism, Dynamical System approach to cognition and contemporary Anticipatory Systems Theory. In this way it might be possible to provide a renovated framework to get an intensional definition of EF, that could begin to bridge “the gap between clinical and cognitive approaches” (Snyder et al., 2015). To substantiate my suggestion, I would like to summarize some key notions taken from our particular translational research programme.

Routinely EF is seen as a wide set of neurocognitive processes and abilities which more or less includes (Chan et al., 2008; Vohs and Baumeister, 2011; Barkley, 2012; Goldstein and Naglieri, 2013):

- Reasoning and problem-solving.
- Anticipating, planning and decision-making.
- The ability to sustain attention and resistance to interference.
- Utilization of feed-forward, feedback and multitasking.
- Cognitive flexibility and the ability to deal with novelty.

Noteworthy is that every aspect that has been mentioned in the recent reviewed literature as being fundamental features of EF, can be associated with the main stages of a “functional systems” (FS) operation as it was devised by Anokhin (1974). Accordingly I have introduced an intensional definition of EF (Labra-Spröhnle, 2015, 2016); paraphrasing Anokhin (1974): EF are any of “those specific mechanisms of the functional system which provide for the universal physiological architecture of the behavioral act.” The advantage of this definition is that it coordinates all of those mechanisms with a systemic framework, giving unity and diversity to the concept of EF. The series of operational stages of a FS can be outlined as follows:

1. “Preparation for decision-making (afferent synthesis),
2. Decision making (selection of an action),
3. Prognosis of the action result (generation of acceptor of action result),
4. Generation of the action program (efferent synthesis),
5. Performance of an action,
6. Attainment of the result,
7. Backward afferentation (feedback) to the central nervous system about parameters of the result,
8. Comparison between the result of action and the prognosis” (Anokhin, 1974).

Moreover the inferential processes that drive cognitive processes extend beyond being mere connections between predicates and were defined by Peirce (1901) and Piaget et al. (2013) comprising three kinds of inferences working in a cycle:

1. Abduction, or hypothesis creation; this is a kind of guess regarding the configurations and possible reactions from the environment.
2. Deduction, allows predictions (logical consequences derived from the former guess or hypothesis), regarding the configurations and reactions from the environment.
3. Induction, assess the result of the actions carried out by the agent by comparing the predictions with the actual results.

An isomorphic functional architecture was experimentally discovered by Anokhin (1974) and Bernstein (1967), in various physiologic processes and goal-directed behaviors, showing the ubiquitous character of these processes in living organisms. The core of operations of a FS are equivalent to the inferential cycle described by Peirce and Piaget. Based on this mapping and in the proposed definition of EF, it can be postulated that inferences are at the core of EF, playing the elusive role of the “executive”; forming an integrative, distributed and hierarchical control with the cognitive functions at the top.

Methodologically, the inferential dynamics can be rendered using diagrams (Labra Sprohnle et al., 1997). The further description of these diagrams can be accomplished using morphological descriptors, i.e., Euclidean and non-Euclidean geometric measures. By these means the EF modeling is achieved by using a multivariate set of geometrical measures and supervised machine learning techniques (Labra-Spröhnle, 2015, 2016).

## Conflict of interest statement

The author declares that the research was conducted in the absence of any commercial or financial relationships that could be construed as a potential conflict of interest. The reviewer Joseph Etherton and handling Editor declared their shared affiliation, and the handling Editor states that the process nevertheless met the standards of a fair and objective review.
